# Participants’ Perceptions on the Use of Wearable Devices to Reduce Sitting Time: Qualitative Analysis

**DOI:** 10.2196/mhealth.7857

**Published:** 2018-03-31

**Authors:** Michelle Takemoto, Brittany Lewars, Samantha Hurst, Katie Crist, Camille Nebeker, Hala Madanat, Jeanne Nichols, Dori E Rosenberg, Jacqueline Kerr

**Affiliations:** ^1^ Department of Family Medicine and Public Health University of California, San Diego La Jolla, CA United States; ^2^ Graduate School of Public Health San Diego State University San Diego, CA United States; ^3^ Kaiser Permanente Washington Health Research Institute Seattle, WA United States

**Keywords:** wearable devices, qualitative research, focus groups, technology

## Abstract

**Background:**

Recent epidemiological evidence indicates that, on average, people are sedentary for approximately 7.7 hours per day. There are deleterious effects of prolonged sedentary behavior that are separate from participation in physical activity and include increased risk of weight gain, cancer, metabolic syndrome, diabetes, and heart disease. Previous trials have used wearable devices to increase physical activity in studies; however, additional research is needed to fully understand how this technology can be used to reduce sitting time.

**Objective:**

The purpose of this study was to explore the potential of wearable devices as an intervention tool in a larger sedentary behavior study through a general inductive and deductive analysis of focus group discussions.

**Methods:**

We conducted four focus groups with 15 participants to discuss 7 different wearable devices with sedentary behavior capabilities. Participants recruited for the focus groups had previously participated in a pilot intervention targeting sedentary behavior over a 3-week period and were knowledgeable about the challenges of reducing sitting time. During the focus groups, participants commented on the wearability, functionality, and feedback mechanism of each device and then identified their two favorite and two least favorite devices. Finally, participants designed and described their ideal or dream wearable device. Two researchers, who have expertise analyzing qualitative data, coded and analyzed the data from the focus groups. A thematic analysis approach using Dedoose software (SocioCultural Research Consultants, LLC version 7.5.9) guided the organization of themes that reflected participants’ perspectives.

**Results:**

Analysis resulted in 14 codes that we grouped into themes. Three themes emerged from our data: (1) features of the device, (2) data the device collected, and (3) how data are displayed.

**Conclusions:**

Current wearable devices for increasing physical activity are insufficient to intervene on sitting time. This was especially evident when participants voted, as several participants reported using a “process of elimination” as opposed to choosing favorites because none of the devices were ideal for reducing sitting time. To overcome the limitations in current devices, future wearable devices designed to reduce sitting time should include the following features: waterproof, long battery life, accuracy in measuring sitting time, real time feedback on progress toward sitting reduction goals, and flexible options for prompts to take breaks from sitting.

## Introduction

Sedentary behavior (SB) is defined as a range of human behaviors that result in an energy expenditure of no more than 1.5 times resting energy expenditure and are typically associated with time spent sitting, reclining, or lying down during waking hours [1-3]. Recent epidemiological evidence indicates that, on average, adults spend approximately 6 hours per day sedentary [4], and older adults are sedentary for approximately 9 hours per day [5]. The fact that individuals are sitting more is problematic because epidemiological studies have found deleterious effects of prolonged SB, including increased risk of cancer, metabolic syndrome, heart disease, and mortality [3,6-9]. Importantly, the negative health outcomes from increased SB are separate from participation in physical activity (PA) [6,7,9].

On the basis of the clear positive benefits associated with increased PA [10], decades of previous research have identified goal setting and self-monitoring as successful intervention strategies to increase PA [10]. Technology as an intervention tool has been used effectively in PA research [11-14]. Pedometers are a powerful change tool that can motivate individuals to increase PA [15-17]. Pedometers are helpful tools in that they allow participants to self-monitor behavior by tracking the number of steps taken throughout the course of the day [15,18]. Additionally, new wearable devices such as Fitbits are based on the same principles as pedometers and combine self-monitoring with individual feedback on progress toward goals [14]. Therefore, wearable devices provide an effective strategy for increasing PA by allowing for tailored goal setting and serving as reinforcement to work toward a specific goal [15].

Although wearable devices have been shown to be effective strategies for increasing PA [14,15], it is unclear how this technology might be applied to SB. One of the many challenges associated with changing SB is the sheer volume of sitting time individuals accumulate throughout the day [2,19]. On the basis of the continuous exposure to the behavior, trying to measure how much time individuals spend sitting can be extremely challenging [20]. Therefore, regular monitoring via technology to reduce participant burden may be an especially valuable intervention tool.

Given the recent surge in epidemiological and laboratory studies highlighting the association between excessive SB and poor health outcomes [6,7,21-24], new interventions to reduce sitting time are necessary. Recent research has explored different methods to interrupt sitting through increased prolonged standing or adding additional sit-to-stand transitions, which are brief postural changes from a seated position into a standing position and back to a seated position. These transitions break up long bouts of sitting and continually interrupt sitting time throughout the course of the day. One potential strategy to interrupt sitting by providing prompts to stand or add transitions is through smartphone apps. Several studies have capitalized on the surge in smartphone apps focusing on health-related outcomes, including PA and SB [25-27].

Just-in-time intervention strategies provide participants with real time feedback regarding their activity; however, a limitation of smartphone apps ability to change sitting time is the likelihood of misclassifying standing as inactivity based on a phone’s location. If a participant puts the phone on a desk while he or she takes a standing break, the accelerometer in the phone would fail to capture this behavior as standing and would instead classify it as inactivity. For a participant working toward a goal to reduce sitting through increased standing, this misclassification can be frustrating and may demoralize his or her motivation to work toward accomplishing the goal. Current wearable devices that focus on prompting participants to move more are not designed to reduce sitting time [28]; however, these devices could be repurposed to target sitting reduction. For future devices, it would be especially valuable if developers could overcome these measurement limitations given the difficulty for individuals to monitor sitting time and the ubiquitous nature of the behavior.

Therefore, a wearable device that tracks accumulated sitting time and prompts behavior change throughout the course of the day (eg, vibration and alarms) could be an especially effective intervention tool [15,17,18]; however, more information on participants’ perspectives toward these devices is needed. To gather end-user feedback on specific products, marketing and advertising companies use focus groups. The benefit of focus groups over one-on-one interviews is that the group setting promotes spontaneous discussion between participants that is not possible in an individual interview [29,30]. Given the rapid innovations in wearable technology combined with the negative health outcomes associated with prolonged sitting, this study used a focus group methodology to explore the perceived usability and acceptability of current wearable devices for SB. The purpose was to better understand if it would be feasible and appropriate to incorporate wearable devices in a 6-month SB study as an intervention tool.

## Methods

### Overview

The research was guided by a combination of inductive and deductive methods in that data collected were used to describe results related to wearable devices and SB.

### Participants

We recruited participants from a previous SB intervention to participate in the focus groups. The Take a Stand study was a two-arm, randomized, pilot trial funded by the Department of Family Medicine and Public Health at the University of California, San Diego (UCSD). The study tested the feasibility and acceptability of a short-term SB intervention. A full description of the study and the findings have been reported previously [31]. Briefly, 30 participants in the age range of 50 to 70 years, with an equal number of workers and nonworkers, were followed for 21 days while the intervention was delivered. The eligibility criteria for participants are shown in Textbox 1.

**Eligibility criteria for participants in the original Take a Stand pilot intervention.**

- Aged 50 to 70 years
- Spent at least an average of 8 hours per day sitting over 5 days
- Able to attend four measurement visits at the University of California, San Diego campus over 4 consecutive weeks
- Willing to wear a thigh-mounted inclinometer 24 hours per day for the entire 21 day study duration
- Able to read and write in English
- Able to provide written informed consent
- Without a serious health condition that would limit their ability to stand

Upon enrollment, participants were randomized to either a decrease sitting or an increase sit-to-stand transition condition. Participants were asked to work on either SB goal over the course of 2 weeks while wearing a thigh-mounted inclinometer called the ActivPAL that objectively measured SB. The device did not provide real time feedback on the behavior, but participants retrospectively viewed the past week’s progress during weekly intervention visits.

Qualitative research focuses on participants who are likely to provide rich information about the specific research questions [32]. Therefore, we used a purposive sampling technique [33] to enroll participants from the Take a Stand study because these individuals had previous experience attempting to change their SB and interacting with the ActivPAL, which is considered a wearable SB device. Therefore, these participants provided feedback that is more informed based on their prior exposure to both SB interventions and devices designed to record activity.

We conducted a total of 4 focus groups in September 2014, and each lasted for 2 hours. The groups were stratified by work status (ie, worker or nonworker) and intervention condition (ie, sit less or increase sit-to-stand transitions). We chose to stratify to elucidate information about how wearable devices might work best depending on the participant’s work status and intervention goal. Previous SB interventions have focused primarily on worksites, and we wanted to explore how participants might favor wearable devices differently depending on work status [34-38]. Additionally, given the novelty of the sit-to-stand transition behavior, we wanted to understand how current wearable devices could be used for this type of behavior. Therefore, we chose to have separate focus groups to reflect the differences we anticipated. There were between 2 and 5 participants per group, depending on participant availability. All participants signed written informed consent and approval was granted by the Human Research Protections Program of the UCSD (Protocol #130817).

### Focus Group Overview

The research team began by identifying wearable devices to include as examples in the focus groups. Current wearable devices focus primarily on PA (ie, steps), but some devices also collect data on SB (ie, inactivity and sitting). We also wanted to include devices that had different wear locations (eg, wrist, back, and thigh) to enhance variability. Participants explored a total of 7 devices with varying features, but all with data on sitting, inactivity, or cues to take breaks from sitting. We defined features as the specific attributes or characteristics of the device (ie, battery life, wear location, and aesthetics). These devices included the ActivPAL, SitFIT, Lumoback, Smart Move shoe insert, Sensoria Sock, Garmin Vivofit, and Jawbone UP (see Table 1).

Given previous research using wearable devices to change PA, we hypothesized that similar devices could be especially effective tools to help reduce sitting time, and we wanted more information from participants regarding the perceived acceptability of current devices on the market. The focus group moderators (JK and KC) have experience with SB research and were involved in the Take a Stand pilot; JK was the principal investigator, and KC was the project manager. However, neither JK nor KC had prior participant interaction during the intervention study, therefore they were able to serve as moderators who were unfamiliar to the participants to allow participants to be as open as possible.

Before beginning the focus groups, participants provided written informed consent, and the moderators stressed the confidential nature of the discussions. Participants were informed that the discussion would be transcribed in real time via a transcriptionist, used for research purposes only, and would not be accessible to anyone outside the research team. To ensure confidentiality, participants did not use their full names. To encourage open communication of thoughts and ideas, the moderator stressed that the opinions of each participant were important, and there were no right or wrong answers. Upon completion of the focus groups, we thanked participants and provided each individual with US $20 as compensation for their participation.

The purpose of the focus groups was to provide insight on wearable devices for SB to inform a larger SB intervention. The overall focus group framework had the following format for each session: (1) a review of the device’s functionality, (2) question and answer for each specific device, (3) voting on devices, (4) review of interfaces, (5) voting on interfaces, and (6) design the magic device via paper prototyping.

**Table 1:** Description of the seven wearable devices reviewed during the focus groups. We organized the devices based on wear location.

Feature of the device	ActivPAL	SitFIT	LUMOback	Vivofit and Jawbone UP	Sensoria Sock and SmartMove
Wear location	Thigh	Pocket	Lower back	Wrist	Foot and ankle
Feedback display	Paper graphs	Device, smartphone, Web	Smartphone	Smartphone, Web Sync with plug	Smartphone, Web
Frequency of feedback	Delayed until visit with study staff	Real time	Real time	Real time	Real time
Prompt type	Vibration	Vibration	Vibration	Red bar^a^; Vibration^b^	Unknown
Prompt adjustable	✓	✓	✓	✓	Unknown
Tracks sitting	✓	✓	✓	Inactivity	Potentially
Tracks sit to stand	✓	✓	✓		Potentially
Tracks steps	✓	✓	✓	✓	✓
Tracks sleep		✓	✓	✓	
Tracks other	None	None	Posture	Calories, distance	Speed, calories, distance, cadence
Waterproof	With tape and supplies			✓	Washable
Battery life	12 days^b^	Unknown	Up to 7 days	1 year^a^; 7 days^b^	Unknown

^a^Feature of the Garmin Vivofit.

^b^Feature of the Jawbone UP.

The first part of the focus groups focused on perceived wearability and functionality of each device. Each participant received a packet with information about each device. The packet included pictures and descriptions of each specific device. To get started, the moderators introduced each device to the participants, including a brief description (see Table 1), and then gave each participant the opportunity to hold the device and see it up close. The moderators then asked participants to describe any benefits or barriers to using the device for an extended period (ie, 6 months).

The first device discussed was the ActivPAL device, which participants wore for 3 weeks during the previous pilot intervention and had experience using. We then moved on to the remaining 6 devices. Participants were probed with questions to determine which device they thought would be the most likely to help them change SB during the course of a 6-month intervention. Questions included “what do you foresee as the biggest challenge to wearing this device for a long-term intervention?” or “what do you think will be make this device helpful?”

The next section of the focus group focused on the interfaces (ie, the medium used to display data to users) for the current devices. The moderators provided a brief overview about interfaces and how they provide feedback about one’s behavior. Some of these interfaces displayed feedback via a smartphone or computer, and others displayed feedback directly on the device itself. The next section focused on discussing the current interfaces available and identifying the benefits and barriers to each. Sample questions included “which do you like the most and why?” and “what do you like least about this interface?” After discussing each interface, participants rated their most and least favorite interfaces.

After having the opportunity to discuss each device, participants had the opportunity to vote on the devices. Specifically, when voting, participants identified their two favorite and two least favorite devices based on their individual preferences. During the final part of the focus group, participants designed their ideal device. This ideal device incorporated the best and worst parts of each of the previously described devices and interfaces, but it could also include elements that do not exist in these devices that would be essential to help individuals reduce their sitting time or increase sit-to-stand transitions. Participants used their creativity to sketch a prototype of the device and describe how it would work. When designing the focus group protocol, we consulted with a colleague who specializes in human computer interaction research, which is the study of how people interact with computers and other technology [39]. The voting and device design sections of the focus group were based on previous work with user experience design, which emphasizes involving the end-users in the initial design process to ensure products are developed that fit user needs [39]. Additionally, we purposely maintained a small number of participants per focus group to ensure that participants had many opportunities to interact with each of the seven devices and participate fully in the voting and design portions. Similar to product testing with consumer companies, we recruited a smaller number of informed participants per group to collect detailed information about the perceived usability and acceptability of the devices.

### Data Analyses

A transcriptionist who was present during the entirety of each session transcribed the focus groups in real time. This methodology is especially effective for focus groups in which participants are encouraged to discuss an experience or process and provide feedback on concrete elements such as aesthetics or ease of use [30]. To facilitate transcription, participants sat behind numbered placards, allowing the transcriptionist to note who was speaking. Two researchers (MT and BL), who have experience coding qualitative data and had worked on the Take a Stand study, analyzed the transcripts. MT completed her doctoral degree at UCSD and has formal training in qualitative and mixed-methods research. BL earned a Master’s degree in Public Health and has experience with qualitative research methods. MT developed the focus group guide, and BL served as a device expert during 2 of the focus groups. Neither MT nor BL were involved in the moderation of the focus groups.

A thematic approach guided data analysis and data were organized into themes that reflected participants’ perspectives. All analyses were done using Dedoose software. First, each coder read the transcripts independently to familiarize themselves with the content. During the second read through, each coder took notes and highlighted significant passages. The first transcript was coded in Dedoose, together by MT and BL, to create an initial codebook. Segments of the content with similar meaning were assigned to the same code. The remaining transcripts were used to refine the concepts of the initial codebook and combine the codes into key themes. When new codes or themes emerged, the codebook was revised, and the previous transcripts were recoded. Because coding occurred in tandem, any discrepancies were resolved in real time and ensured that all transcripts were coded by both researchers. Coding occurred over the course of several months, and saturation was reached when no new codes were generated after a final review of the transcripts. Key quotes were selected that were representative of the main themes.

## Results

### Participants

A total of 15 people participated across the 4 focus groups, with the two largest groups having 5 participants and the smallest group, consisting of nonworkers from the sit-to-stand transition intervention condition, having 2 participants. The average age was 59 years, and 87% (13/15) were female (see Table 2). The majority (12/15, 80%) were white, non-Hispanic, and there was an almost equal distribution between work status and intervention condition (8/15, 53%). From the 14 codes analyzed, 3 overall themes emerged related to the pros and cons associated with different aspects of the devices: (1) features of the device, (2) data the device collects, and (3) how data are displayed. Please see Multimedia Appendix 1 for a full description of the codes with definitions and seminal quotes.

### Features of the Device

Participants reported mixed feelings about the various features of each device. Some participants liked devices that were directly adhered to the body because they were never forced to remember to put on the device; however, other participants commented that they would not wear an adhered device long-term (eg, ActivPAL). Participants were concerned about the pocket-worn SitFit device because, as one participant described, “most of the pants I wear don’t have pockets.” They would be more likely to use the device if they could attach it to a belt that they could wear with all pants. However, other participants had no concerns with the pocket placement and could easily incorporate it into their daily lives.

Aesthetics of the device were important both for device look (ie, did the device come in different colors [eg, Jawbone and VivoFit]) and for how the device would fit into an everyday routine. For example, participants struggled to understand how they could incorporate the Sensoria sock device or SmartMOVE shoe insole into everyday routines because not all outfits required socks or tennis shoes. One participant wore “sandals all the time,” and another participant reported being “barefoot most of the time,” which meant the form and location of these devices would make it challenging to wear consistently. Although this was likely a San Diego warm weather bias and might not be an issue in other areas with different climate.

During the dream device design portion, participants ideally wanted a wear location that could be flexible depending on what they needed for specific days. For example, one participant stated:

My ideal device would be kind of adjustable, depending on what you're going to wear and maybe on your back one day or your leg...whether that be [adhered with] some kind of adhesive...or a belt so it can be interchangeable.

**Table 2:** Descriptive statistics for participants in the focus groups (N=15).

Characteristic		Value
Age (years), mean (SD)		59 (6.21)
**Gender, n (%)**		
	Female	13 (87)
**Race, n (%)**		
	White, non-Hispanic	12 (80)
**Condition, n (%)**		
	Sit less	8 (53)
**Work status, n (%)**		
	Full-time employed	8 (53)

Feedback was important, and participants wanted control over how often they received the feedback. Most participants requested real time feedback (eg, Jawbone UP and SitFit) as a method to actively work toward the goal throughout the course of the day. Prompts were another desired feature, and again, participants wanted control over the type of prompt (ie, vibration [Jawbone] and visual [Vivofit]), and frequency (ie, ability to deactivate prompts during sleep hours or change prompts depending on work schedule). When designing the dream device, participants emphasized the importance of programmability to allow everyone to choose feedback and prompts that were the most relevant and helpful to them as individuals. A participant said, “the frequency of the feedback would be programmable by the individual.”

Participants mentioned practical concerns such as battery life and waterproofing. Longer battery life (eg, Vivofit) was a benefit for several participants as it eliminated the need to remember to charge the device frequently. Finally, whether or not a device was waterproof (eg, Vivofit) and could be worn in the shower, thereby not requiring participants to remove the device and subsequently remember to put the device back on (eg, Jawbone, Lumoback, and Sitfit), impacted participants’ willingness to use the device long-term. When describing their dream devices, participants highlighted the importance of these practical features of the device when designing a device for long-term use.

### Data the Device Collects

Participants were concerned about device accuracy to detect sitting time and preferred devices that provided information on sitting time as opposed to inactivity. As mentioned previously, most current wearable devices focus on inactivity and thereby classify both sitting and standing as inactivity (eg, Jawbone and Vivofit). However, other devices (eg, ActivPAL, SitFit, and Lumoback) are specifically designed to measure sitting and standing as separate, and participants favored devices able to distinguish between these distinct behaviors. Additionally, some participants doubted a device’s accuracy based on the device’s wear location (eg, pocket where the SitFit was worn or wrist where the Vivofit and Jawbone were worn were seen as less accurate). As one participant described it when designing the dream device:

...it has to track sitting. It has to track sitting to standing based on the goal.

Participants had mixed reactions to the amount of information different devices collected. For example, some people liked the idea of collecting additional information (eg, sleep, posture, and calories), whereas other people were concerned that by collecting more information, there would be more opportunity to question the accuracy of the data collected. One participant stated:

There’s more to question when you get a lot of data...If it thinks that I’m driving three hours, but I really only drive one hour but I rode my bicycle 2 hours, and it’s confusing bicycling with driving, I might say to myself, oh, this isn’t accurate...I will lose confidence with the accuracy of the device.

Devices that were not able to detect sit-to-stand transitions (eg, Jawbone UP and Vivofit) were viewed less favorably by participants from the sit-to-stand transition condition. Control over data, which allowed participants to choose how the data are displayed and who can access the data, was a priority, with one participant stating:

I’d rather have control..., even [if the device is] not comfortable, than no control over something like data.

### How Data Are Displayed

Participants had varying opinions on where and how to display the data. Some participants liked data displayed on a smartphone (eg, Jawbone and Lumoback), whereas others were adamantly against it because they did not own a smartphone and had no plans to purchase one anytime soon. One individual talked about the need to “get away from the phone,” which was a barrier to any device tied to a smartphone display. Participants also liked the idea of displaying long-term data on a computer to allow them to see “the progression of change over time.” Frequency of feedback also varied as some participants wanted to see progress throughout the course of the day, whereas others would only want to see the data every few days or weekly.

Whatever medium was used, participants wanted the data displayed to be specific to SB. Participants viewed the devices that only displayed information related to activity or inactivity less than ideal, given the focus on SB. Participants preferred interfaces with data displayed in a way that was “easy to understand”; provided a quick summary of overall behaviors; used a combination of graphs, charts, and images; and were visually appealing. Additionally, if the interface used colors to represent behaviors, participants commented that the colors should be intuitive. For example, if they were focusing on reducing sitting with standing, time spent sitting should be highlighted in red, and standing should be represented with green. One of the featured interfaces had reversed these colors, and participants felt this was counterintuitive and confusing. As described by one participant, “it’s very dumb.” On the contrary, interfaces that had a lot of information with small font, a busy display, and required “too much reading” were viewed negatively. Flexibility was highlighted again when participants were designing dream devices, as participants emphasized that “everybody is different,” and being able to modify how the data are presented would be a key feature of the ideal wearable device.

### Voting

Across the 4 focus groups, the most popular device was tied between the SitFit and the Jawbone UP or Vivofit, with 11 favorite votes for each, and the least popular device was the Sensoria Sock or SmartMove, with 10 least favorite votes, and the Lumoback received 8 least favorite votes. One theme that arose from this portion of the focus group was that participants had a difficult time choosing favorites because none of the devices were perfect. One participant stated, “I was sort of doing a process of elimination more than I was activity voting for the favorites.”

However, the votes reflected the themes because the Sitfit and Jawbone UP or Vivofit were specific to detecting sitting time, did not require charging, and had prompting capabilities. Although the Sensoria Sock or SmartMove and Lumoback were good at detecting sitting versus standing or posture, the wear location was not conducive for long-term use.

Although participants continued to mention accuracy as an important component during the device review section of the focus group, when they were asked to vote on their most and least favorites, the devices that may have been better at detecting sitting versus standing, but had less than optimal wear locations were viewed less favorably. Given these results, participants seemed more willing to trade on accuracy if it meant they could easily incorporate the device into their daily routines. These results emphasize the need to develop wearable devices that are not only able to distinguish between behaviors, but can be easily incorporated into participants’ lives to promote long-term use and sustainability.

## Discussion

### Principal Findings

As the evidence around the negative health effects associated with increased SB continues to emerge, interventions to reduce this behavior are becoming increasingly important. Wearable devices represent a novel method to intervene on sitting time, given their numerous capabilities that aid in behavior change, including real time feedback and prompts to interrupt the behavior. However, there has been limited research highlighting the perceived usability and acceptability of these devices to change SB in interventions. Furthermore, most of the current devices on the market emphasize PA as a primary focus and encourage movement. Therefore, it is unclear how to incorporate these devices into SB research. This study explored the barriers and benefits associated with existing wearable devices to reduce sitting time using feedback directly from participants who have intimate experience trying to change this behavior.

Overall, participants were amenable to using wearable devices to change behavior; however, a major limitation of the current devices available was the focus on movement or inactivity as opposed to sitting or standing [28,40]. Additionally, few devices collected information on sit-to-stand transitions or provided feedback related to this behavior. As one participant described, “...by not focusing on sitting time, the device would fail to get a reduction in sitting time.”

Given that participants frequently commented that feedback is a critical component necessary to change behavior, devices that do not provide feedback on the specific behavior, either sitting or sit-to-stand transitions, would not be effective.

Another key finding is that flexibility across all features (eg, wear location, prompting, and feedback) is essential. A common theme across all focus groups was that everybody is different. For example, some participants thought a wrist-worn device would fit perfectly into their daily routine, whereas others would never wear such a device. Some participants only wanted to view data via a mobile phone, whereas other participants would never view data on their phone. Additionally, practical features of devices (eg, waterproof and battery life) were especially important. Therefore, the design of future wearable devices for SB should highlight flexibility and functionality as much as possible to strengthen buy-in from users.

Our study is not without limitations. Specifically, the sample size was small and the majority of participants were female; white, non-Hispanic. Ideally, focus groups should be larger (ie, more than 4 participants per group), and therefore, the group with only 2 participants was especially small given traditional standards. However, their experience from the previous pilot intervention enabled them to have a more informed perspective on the barriers and benefits to using wearable devices to reduce sitting time, which attenuated this limitation. Also given the interactive nature of the focus groups, we purposely chose to limit number of participants to allow for a more thorough exploration into each device.

Another limitation was that participants only had experience using the ActivPAL device for an extended period of time, which does not provide real time feedback and did not have the opportunity to try the other wearable devices. Therefore, participants may have had better perceptions toward the ActivPAL based on simple familiarity with the device and may not have been able to comment fully on the ability for a device to provide real time feedback given their limited experience with this feature. Future research could have participants try each device for an extended period to get more information on how the device may or may not fit into the everyday routine.

Finally, although we stratified by work status and intervention condition, the themes were consistent across focus groups, which could be because of the fact that the two smallest focus groups consisted of participants from the sit-to-stand transition intervention condition. Future studies could expand upon this. The strengths of our study include the use of qualitative methods to gain more insight into the feasibility of using wearable devices to reduce sitting time.

### Conclusions

Evidence shows that excessive SB is unhealthy. Wearable devices represent a novel intervention tool for SB that has the potential for large-scale dissemination and impact. Previous research on a variety of health behaviors (eg, PA, diet) has found that self-monitoring is a key construct for behavior change [41,42]. Currently, there is no self-monitoring tool for sitting. Given the ubiquitous nature of the behavior and the fact that society at large is becoming even more sedentary [8,43], new research into effective self-monitoring tools is necessary. Without proper tools to self-monitor behavior, individuals will continue to struggle to self-assess, which could make behavior change even more challenging. Overall, participants viewed wearable devices as usable and acceptable; however, current models on the market lack a specific focus on SB and are thereby inefficient in targeting behavior change. In light of these challenges, new research that specifically addresses SB is needed to push the field forward. Given the high variability in desired features, feedback, and wear location, research involving the end-user in the design is not only recommended, but should be required.

## Appendix

Multimedia Appendix 1 Themes or codes and seminal participant quotes.

## Abbreviations

PA: physical activity
SB: sedentary behavior
UCSD: University of California, San Diego
