# Supplementary material for: Participants’ Perceptions on the Use of Wearable Devices to Reduce Sitting Time: Qualitative Analysis
Source: JMIR Mhealth Uhealth. 2018 Mar 31;6(3):e73. doi: 10.2196/mhealth.7857 (PMC5897621; doi:10.2196/mhealth.7857)
Supplement: Multimedia Appendix 1 [file mhealth_v6i3e73_app1.pdf]

Appendix 1. Themes or codes and seminal participant quotes.

Theme	Code	Definition	Seminal pro quote	Seminal con quote
Theme 1: features of the device	Battery life	How long the device is able to work without charging; how is the device charged	*And I also like the fact this one has a year battery as opposed to this one which is seven days.*	*And it's just I like not having to worry about plugging them in weekly and whatever. Something where every night you'd have to remember to plug it in and that's a pain.*
	Comfort	How comfortable the device would be to wear (ie, size of device, bulk, weight, and how the device is affixed to the body)	*The tape didn't bother me, you know, it was comfortable.*	*I'm not wearing this piece of rubber. It would be real uncomfortable.*
	Wear location	Where the device is worn	*And I'm better with things that are stuck to my body. I don't have to deal with it or remember it.*	*My pockets on shallow. My lipstick, my Kleenex. I probably could get it in the other pocket, but see I'd be really worried that I'd lose it.*
	Aesthetics	How the device looks (ie, color)	*I like the fact that it's got different colors. I like the black.*	
	Waterproof	Whether or not the device is waterproof	*I'm glad to hear that it's waterproof. Because I always*	*My only problem would be I go to the ocean all the time.*

			have my hands in water so it seems like they're always getting wet.	*Would it get in the way of the ocean?*
	Prompts	Whether or not participants would have control over type of prompt (ie, visual vs tactile) and when device prompts (ie, during waking hours)	*And I need that little reminder that I've been sitting too long. Because I read a lot...I can get carried away reading and a good two hours, I haven't moved. So I need a reminder to get up and move.*	*I'm afraid I wouldn't see the red line. I think I would need the vibration or something. When I get business busy, I don't know that I would notice.*
	Feedback	Ability to receive real-time or instant feedback (if desired) and control over frequency of feedback	*That's more helpful than anything to remind me to get up all during day and the evenings. The evening is my biggest problem. So something like that where I saw, well, I have 30 in by the end of the day, but I also need to be thinking about the evening too.*	*There's not really instant feedback that I should have unless you have an iPhone with you.*
Theme 2: data device collects	Accuracy	How accurate the device would be to classify behaviors (ie, sitting and standing)	*So long as it's accurate. That would be great.*	*You know, I could run three miles just in a conversation with my hands. It detects your hand movement.*
	Control over data	Who owns the date (ie, University of California,	*I'd rather have control over something, even though it's not*	*The Garmin, I really, really, like. I just—I don't like other*

		San Diego vs private company) and whether or not participants or study have control over how the data are displayed and reviewed	*comfortable than no control over something like data.*	*countries being able to track my data*.
	Information device collects	What type of data device collects or measures (ie, sitting, standing, stepping, sit-to-stand transitions, and calories)	*That's nice that it incorporates both of them, the steps and the sitting cause I've worn a pedometer for a long time for another study and I just kept wearing it.*<br>*It would be nice to have more things incorporated into one. If you're going to have to keep up with something, have as much info as you can.*	*The whole sitting for periods of time is kind of what attracted me to this so if you don't have a way to track that it's like—probably wouldn't buy one.*
Theme 3: how data are displayed	Data displayed on device	Device itself features a data display	*That's more helpful to than anything to remind me to get up all during day and the evenings. The evening is my biggest problem. So something like that where I saw, well, I have 30 in by*	*That was actually my first response when I was...pressing the buttons to see the different functions, I can't see [the data]. I know something's there.*

		the end of the day, but I also need to be thinking about the evening too.	
Data displayed on computer	Ability to review data on computer	*I think it's more feasible on a day-to-day basis to see that instant feedback as you're moving throughout the day because you're so busy then to have to go to your phone for something else or your computer. But the long term, when you have time and get to see and track your results, I think [a computer] would be awesome.*	*No [data on] a computer. That's too bad.*
Data displayed on a mobile phone	Ability to review data on a mobile phone	*I would like something that could interface with like an iPad or an iPhone or something like that so I could see—and just see it. I'm just visual so I want that.*	*I am a cave woman. I do not have a smartphone, and I have no immediate plans to buy a smartphone. So something that had an interface or feedback on a smartphone would not work for me.*
Interface	How the information is displayed on the	*Easy that, you know, you can look at it easily and see what's*	*This one I selected, it's too busy, too condensed, and also*

	interfaces (ie, bar graphs, charts, and words) including how helpful the data are for understanding behavior (eg, colors represent behaviors, summary of activities, and ability to quickly understand outcomes)	*going on. It's easy to read.*	*they made a mistake. To me it's very dumb to have [red] stand [for standing] and [green] standing for just the opposite [sitting].*
